# Synthesis and biological evaluation of novel 3-(quinolin-4-ylamino)benzenesulfonamides as carbonic anhydrase isoforms I and II inhibitors

**DOI:** 10.1080/14756366.2019.1652282

**Published:** 2019-08-14

**Authors:** Mohammad M. Al-Sanea, Ahmed Elkamhawy, Sora Paik, Silvia Bua, So Ha Lee, Mohamed A. Abdelgawad, Eun Joo Roh, Wagdy M. Eldehna, Claudiu T. Supuran

**Affiliations:** a Department of Pharmaceutical Chemistry, College of Pharmacy, Jouf University, Sakaka, Saudi Arabia;; b Chemical Kinomics Research Center, Korea Institute of Science and Technology (KIST), Seoul, Republic of Korea;; c Department of Pharmaceutical Organic Chemistry, Faculty of Pharmacy, Mansoura University, Mansoura, Egypt;; d Department of NEUROFARBA, Section of Pharmaceutical and Nutraceutical Sciences, University of Florence, Firenze, Italy;; e Department of Pharmaceutical Organic Chemistry, Beni-Suef University, Beni-Suef, Egypt;; f Division of Bio-Medical Science & Technology, KIST School, Korea University of Science and Technology, Seoul, Republic of Korea;; g Department of Pharmaceutical Chemistry, Faculty of Pharmacy, Kafrelsheikh University, Kafrelsheikh, Egypt

**Keywords:** Benzenesulfonamides, carbonic anhydrase, quinolines, synthesis, cytosolic isoforms hCA I and II

## Abstract

Carbonic anhydrases (CAs, EC 4.2.1.1) are crucial metalloenzymes that are involved in diverse bioprocesses. We report the synthesis and biological evaluation of novel series of benzenesulfonamides incorporating un/substituted ethyl quinoline-3-carboxylate moieties. The newly synthesised compounds were *in vitro* evaluated as inhibitors of the cytosolic human (h) isoforms hCA I and II. Both isoforms hCA I and II were inhibited by the quinolines reported here in variable degrees: hCA I was inhibited with *K*_I_s in the range of 0.966–9.091 μM, whereas hCA II in the range of 0.083–3.594 μM. The primary 7-chloro-6-flouro substituted sulphfonamide derivative **6e** (*K*_I_ = 0.083 μM) proved to be the most active quinoline in inhibiting hCA II, whereas, its secondary sulfonamide analog failed to inhibit the hCA II up to 10 μM, confirming the crucial role of the primary sulphfonamide group, as a zinc-binding group for CA inhibitory activity.

## Introduction

Carbonic anhydrases (CA) (CAs, EC 4.2.1.1) are zinc-containing metalloenzymes that are present in most organisms all over the tree of life^1^^,^^2^. These metalloenzymes efficiently catalyse the rapid interconversion of carbon dioxide and water to bicarbonate and protons. In humans, this fundamental reaction encompasses three simple chemical entities, CO_2_, HCO_3_^−^, and H^+^, essential in a host of physiological and pathological processes, such as calcification, bone resorption, electrolyte secretion, pH and CO_2_ homeostasis, tumorigenicity, and several biosynthetic reactions^3–5^. Eight distinct genetic enzymatic families were identified; the α-, β-, γ-, δ-, ζ-. η-, θ- and ι-CAs^3–5^. To date, 15 human (h) isoforms of CA have been identified, which have all belong to the α-class and have different patterns of tissue distribution and cellular localisation as the following; cytosolic (I, II, III, VII, and XIII), membrane-bound (IV, IX, XII, and XIV), secreted (VI) and mitochondrial (VA and VB) forms^3–5^. CA I and II are present at high concentrations comparing to other CA isoforms in the erythrocytes cytosol and several other tissues.

**Figure 1.**
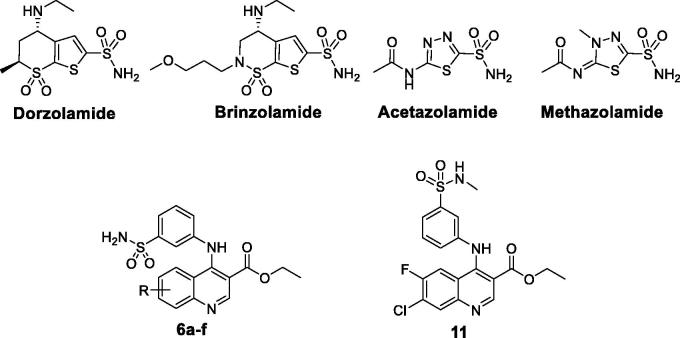
Structures of some approved CAIs antiglaucoma drugs, and the target quinolines **6a–f** and **11**.

Several important pathological consequences result from the dysfunction of hCA II activity, thus this isoform is an established drug target for a multitude of diseases, such as oedema^6^, epilepsy^7^, acute mountain sickness^8^, and glaucoma, where excessive aqueous humour is secreted within the eye, with the subsequent increase in the intraocular pressure (IOP)^9–11^. CA inhibitors (CAIs) are able to diminish IOP by decreasing the rate of bicarbonate formation and thus secretion of the aqueous humour. For more than 60 years, carbonic anhydrase inhibitors are in clinical use for the treatment of glaucoma, such as the topically acting dorzolamide and brinzolamide drugs, and the systemic acetazolamide and methazolamide drugs^9^ (Figure 1).

Pertaining to its prevalence in diverse natural products, such as alkaloids, and in different pharmacologically active substances, quinoline stands out as a promising privileged scaffold that is endowed with a wide spectrum of biological activities. Just to name a few, antimalarial^12^, antileishmanial^13^, anti-tubercular^14^, antidepressant^15^, anticancer^16^^,^^17^ and antiglaucoma^18^ actions were reported for quinoline derivatives. Accordingly, medicinal chemists embarked on exploring various quinoline-based molecules comprehending their potential to develop promising and efficient bioactive compounds^19^^,^^20^. These efforts led to FDA approval for several quinoline-based drugs such as the anticancer agent lenvatinib, the anti-asthmathic drug montelukast, the antiviral Clioquinol, and the anaesthetic Dibucain.

In the present study, we report a new series of primary benzenesulfonamides incorporating un/substituted ethyl quinoline-3-carboxylate (**6a–6f**, Figure 1) as well as the secondary benzenesulfonamide analogue (**11**, Figure 1), with the prime goal of developing effective quinoline-based antiglaucoma candidates targeting the cystolic isoform hCA II. These quinoline-based benzenesulfonamides were evaluated *in vitro* for their inhibitory activity towards the physiologically relevant hCA isoforms I and II, using stopped-flow CO_2_ hydrase assay.

## Materials and methods

### Chemistry

All reaction and manipulations were performed in nitrogen atmosphere using standard Schlenk techniques. All reaction solvents and reagents were purchased from commercial suppliers and used without further purification. Microwave-assisted synthesis was carried out in a Biotage Initiator + apparatus operating in single mode, the microwave cavity producing controlled irradiation at 2.45 GHz (Biotage AB, Uppsala, Sweden). The reactions were run in sealed vessels. These experiments were performed by employing magnetic stirring and a fixed hold time using variable power to reach (during 1 − 2 min) and then maintain the desired temperature in the vessel for the programed time period. The temperature was monitored by an IR sensor focused on a point on the reactor vial glass. The IR sensor was calibrated to internal solution reaction temperature by the manufacturer. The NMR spectra were obtained on Bruker Avance 400 (400 MHz ^1^H and 101 MHz ^13 ^C NMR). ^1^H NMR spectra were referenced to tetramethylsilane (δ = 0.00 ppm) as an internal standard and were reported as follows: chemical shift, multiplicity (b = broad, s = singlet, d = doublet, t = triplet, dd = doublet of doublet, m = multiplet). Column chromatography was performed on Merck Silica Gel 60 (230–400 mesh) and eluting solvents for all of these chromatographic methods were noted as appropriated-mixed solvent with given volume-to-volume ratios. TLC was carried out using glass sheets pre-coated with silica gel 60 F_254_ purchased by Merk. High-resolution spectra were performed on Waters ACQUITY UPLC BEH C18 1.7 μ–Q-TOF SYNAPT G2-Si High Definition Mass Spectrometry. Compounds **3a-f**, **4a-f**^21–22^ and **10**^23^ were previously prepared.

### *General procedure for preparation of compounds* 4a–f

A solution of compounds **3a**–**f** (1.0 mmol) in POCl_3_ (6 ml) was refluxed for 1 h. The mixture was evaporated in *vacuo* and the residue was extracted with methylene chloride, crushed ice and aqueous NH_3_. The organic layer was dried over Na_2_SO_4_ and concentrated. The residue was purified by column chromatography (SiO_2_, ethyl acetate (EA): *n*-Hex 10: 1) to get key intermediates **4a-f**^21^^,^^22^.

### *General procedures for preparation of the target quinolines* 6a–f and 11

To a MW vial, were successively added the appropriate ethyl 4-chloroquinoline-3-carboxylate derivative **4a-f** (0.21 mmol), 3-aminobenzenesulfonamide **5** (0.036 gm, 0.21 mmol) or 3-amino-*N*-methylbenzenesulfonamide **10** (0.040 gm, 0.21 mmol), and ethanol (12 ml) at room temperature. The MW vial was sealed and heated under MW conditions for 30 min at 150 °C. The mixture was evaporated *in vacuo* and the residue was extracted with EA and NaHCO_3_ (aq). The organic layer was dried over Na_2_SO_4_ and concentrated. The residue was purified by column chromatography (SiO_2_, EA: *n*-Hex), in a gradient elution with 1:5 (EA: n-hex) ratio, to furnish quinolines **6a–f** and **11**, respectively.

### *Ethyl 4-((3-sulphamoylphenyl)amino)quinoline-3-carboxylate* (6a)

White solid, yield: 49%, mp: 183.6 − 185.0 °C; ^1^H NMR (DMSO-*d*_6_, 400 MHz) *δ ppm*: 1.16 (t, *J* = 6.8 Hz, 3H, CH_2_ CH_3_), 4.05 (*q*, *J* = 6.8 Hz, 2H, CH_2_CH_3_), 7.16–7.18 (*m*, 1H, H-2 of benzenesulfonamide), 7.34 (*s*, 2H, SO_2_NH_2_), 7.45–7.56 (*m*, 4H, H-4,5,6 of benzenesulfonamide and H-6 quinoline), 7.80–7.84 (*m*, 1H, H-7 quinoline), 8.01 (d, *J* = 8.0 Hz, 1H, H-5 quinoline), 8.10 (d, *J* = 8.4 Hz, 1H, H-8 quinoline), 9.01 (*s*, 1H, H-2 quinoline), 9.75 (*s*, 1H, NH); ^13 ^C NMR (DMSO-*d*_6_, 101 MHz) *δ ppm*: 14.31 (CH_3_), 61.48, 111.07 (quinoline C-3), 116.42 (benzenesulfonamide C-2), 119.82 (benzenesulfonamide C-4), 121.40 (quinoline C-10), 121.92 (benzenesulfonamide C-6), 124.88 (quinoline C-5), 126.55 (quinoline C-6), 130.05 (quinoline C-8), 130.22 (benzenesulfonamide C-5), 131.95 (quinoline C7), 144.44 (benzenesulfonamide C-3), 145.60 (quinoline C-4), 148.12 (benzenesulfonamide C-1), 150.36 (quinoline C-2), 151.38 (quinoline C-9), 166.81 (C = O); HRMS (ESI) for C_18_H_18_N_3_O_4_S: calcd 372.1018, found: 372.1017 [M + H]^+^.

### *Ethyl 6-methyl-4-((3-sulphamoylphenyl)amino)quinoline-3-carboxylate* (6 b)

Yellow solid, yield: 97%, mp: 223.0 − 224.5 °C; ^1^H NMR (DMSO-*d*_6_, 400 MHz) *δ ppm*: 1.09 (*t*, *J* = 5.6 Hz, 3H, CH_2_ CH_3_), 2.40 (*s*, 3H, CH_3_), 3.89 (*q*, *J* = 5.6 Hz, 2H, CH_2_CH_3_), 7.25 (*s*, 1H, H-2 of benzenesulfonamide), 7.39 (*s*, 2H, SO_2_NH_2_), 7.47–7.55 (*m*, 3H, H-4,5,6 of benzenesulfonamide), 7.73–7.74 (*m*, 1H, H-5 quinoline), 7.96 (*s*, 1H, quinoline H-7), 8.26 (*s*, 1H, H-8 quinoline), 8.88 (*s*, 1H, H-2 quinoline), 10.25 (*s*, 1H, NH); ^13 ^C NMR (DMSO-*d*_6_, 101 MHz) *δ ppm*: 14.22 (CH_2_ CH_3_), 21.73 (CH_3_), 61.57 (CH_2_), 111.34 (quinoline C-3), 116.98 (benzenesulfonamide C-2), 120.58 (quinoline C-10), 121.17 (benzenesulfonamide C-4), 122.55 (benzenesulfonamide C-6), 123.97 (quinoline C-6), 127.10 (quinoline C-5), 130.19 (benzenesulfonamide C-5), 134.72 (quinoline C-8), 136.89 (quinoline C-7), 143.66 (benzenesulfonamide C-3), 145.60 (benzenesulfonamide C-1), 144.74 (quinoline C-2), 148.89 (quinoline C-9), 166.02 (C = O); HRMS (ESI) for C_19_H_20_N_3_O_4_S: calcd 386.1175, found: 386.1170 [M + H]^+^.

### *Ethyl 6-methoxy-4-((3-sulphamoylphenyl)amino)quinoline-3-carboxylate* (6c)

White solid, yield: 61%, mp: 214.9 − 216.3 °C; ^1^H NMR (DMSO-*d*_6_, 400 MHz) *δ ppm*: 1.13 (*t*, *J* = 6.8 Hz, 3H, CH_2_ CH_3_), 3.73 (*s*, 3H, OCH_3_), 3.99 (*q*, *J* = 6.8 Hz, 2H, CH_2_CH_3_), 7.14–7.16 (*m*, 1H, H-2 of benzenesulfonamide), 7.33 (*s*, 2H, SO_2_NH_2_), 7.42–7.48 (*m*, 4H, H-4,5,6 of benzenesulfonamide and H-7 quinoline), 7.96 (*s*, 1H, H-5 quinoline), 7.91–7.93 (*m*, 1H, H-8 quinoline), 8.84 (*s*, 1H, quinoline H-2), 9.95 (*s*, 1H, NH); ^13 ^C NMR (DMSO-*d*_6_, 101 MHz) *δ ppm*: 14.31 (CH_2_ CH_3_), 21.73 (CH_3_), 55.92 (OCH_3_) 61.40 (CH_2_), 103.51 (quinoline C-4), 111.54 (quinoline C-3), 116.36 (benzenesulfonamide C-2), 119.51 (benzenesulfonamide C-4), 121.78 (quinoline C-10), 122.35 (benzenesulfonamide C-6), 123.78 (quinoline C-7), 130.17 (benzenesulfonamide C-5), 131.56 (quinoline C-8), 144.36 (benzenesulfonamide C-3), 145.55 (quinoline C-9), 146.16 (benzenesulfonamide C-1), 146.59 (quinoline C-2), 148.90 (quinoline C-4), 157.51 (quinoline C-6), 166.90 (C = O); HRMS (ESI) for C_19_H_20_N_3_O_5_S: calcd 402.1124, found: 402.1126 [M + H]^+^.

### *Ethyl 6-bromo-4-((3-sulphamoylphenyl)amino)quinoline-3-carboxylate* (6d)

Yellow solid, yield: 71%, mp: 235.6 − 237.2 °C; ^1^H NMR (DMSO-*d*_6_, 400 MHz) *δ ppm*: 1.07 (*t*, *J* = 5.6 Hz, 3H, CH_2_ CH_3_), 3.88 (*q*, *J* = 5.6 Hz, 2H, CH_2_CH_3_), 7.17 (*s*, 1H, H-2 of benzenesulfonamide), 7.36 (*s*, 2H, SO_2_NH_2_), 7.45–7.51 (*m*, 3H, H-4,5,6 of benzenesulfonamide), 7.94 (*s*, 2H, H-7,8 quinoline), 8.53 (*s*, 1H, H-5 quinoline), 8.92 (*s*, 1H, H-2 quinoline), 9.71 (*s*, 1H, NH); ^13 ^C NMR (DMSO-*d*_6_, 101 MHz) *δ ppm*: 14.22 (CH_3_), 61.49 (CH_2_), 111.61 (quinoline C-3), 116.40 (benzenesulfonamide C-2), 119.81 (quinoline C-10), 119.95, (benzenesulfonamide C-4), 121.47 (benzenesulfonamide C-6), 123.08 (quinoline C-6), 126.60 (quinoline C-5), 130.21 (benzenesulfonamide C-5), 132.13 (quinoline C-8), 134.76 (quinoline C-7), 144.01 (benzenesulfonamide C-3), 145.68 (benzenesulfonamide C-1), 146.37 (quinoline C-2), 148.91 (quinoline C-9), 152.06 (quinoline C-4), 166.33 (C = O); HRMS (ESI) for C_18_H_17_BrN_3_O_4_S: calcd 450.0123, found: 450.0127 [M + H]^+^.

### *Ethyl 7-chloro-6-fluoro-4-((3-sulphamoylphenyl)amino)quinoline-3-carboxylate* (6e)

Yellow solid, yield: 55%, mp: 190.0 − 191.0 °C; ^1^H NMR (DMSO-*d*_6_, 400 MHz) *δ ppm*: 1.08 (*t*, *J* = 7.2 Hz, 3H, CH_2_CH_3_), 3.91 (*q*, *J* = 7.2 Hz, 2H, CH_2_CH_3_), 7.18–7.20 (*m*, 1H, H-2 of benzenesulfonamide), 7.45–7.49 (*m*, 3H, H-4,5,6 of benzenesulfonamide), 7.36 (*s*, 2H, SO_2_NH_2_), 8.18 (d, *J* = 11.2 Hz, H-8 quinoline), 8.25 (d, *J* = 7.6 Hz, 1H, H-5 quinoline), 8.91 (*s*, 1H, H-2 quinoline), 9.68 (*s*, 1H, NH); ^13 ^C NMR (DMSO-*d*_6_, 101 MHz) *δ ppm*: 14.22 (CH_3_), 61.58 (CH_2_), 110.16, 110.40 (quinoline C-5), 111.47 (quinoline C-3), 116.52 (benzenesulfonamide C-2), 120.18 (quinoline C-10), 121.24 (benzenesulfonamide C-4), 121.85 (quinoline C-10), 125.21 (benzenesulfonamide C-6), 124.41 (benzenesulfonamide C-5), 130.29 (quinoline C-7), 131.58 (quinoline C-8), 143.76 (benzenesulfonamide C-3), 145.69 (benzenesulfonamide C-1), 147.05, 147.52 (quinoline C-6), 152.27 (quinoline C-9), 153.76 (quinoline C-2), 156.21 (quinoline C-4), 166.26 (C = O); HRMS (ESI) for C_18_H_16_ClFN_3_O_4_S: calcd 424.0534, found: 424.0525 [M + H]^+^.

### *Ethyl 5,7-dichloro-4-((3-sulphamoylphenyl)amino)quinoline-3-carboxylate* (6f)

Yellow solid, yield: 98%, mp: 228.7 − 230.3 °C; ^1^H NMR (DMSO-*d*_6_, 400 MHz) *δ ppm*: 1.21 (*t*, *J* = 7.2 Hz, 3H, CH_2_CH_3_), 4.17 (*q*, *J* = 7.2 Hz, 2H, CH_2_CH_3_), 6.97–6.99 (*m*, 1H, H-2 of benzenesulfonamide), 7.28–7.37 (*m*, 3H, H-4,5,6 of benzenesulfonamide), 7.72 (d, *J* = 6.0 Hz, 1H, H-6 quinoline), 8.08 (*s*, 1H, H-8 quinoline), 9.09 (*s*, 1H, H-2 quinoline), 9.83 (*s*, 1H, NH); ^13 ^C NMR (DMSO-*d*_6_, 101 MHz) *δ ppm*: 14.22 (CH_3_) 62.08 (CH_2_), 114.54 (quinoline C-3), 117.87 (benzenesulfonamide C-2), 118.79 (quinoline C-10), 120.52 (benzenesulfonamide C-4), 127.44 (benzenesulfonamide C-6), 129.58 (quinoline C-6), 130.43 (quinoline C-5, 8), 131.42 (benzenesulfonamide C-5), 136.15 (quinoline C-7), 136.62 (benzenesulfonamide C-1), 145.22 (quinoline C-4), 148.09 (quinoline C-9), 152.40 (quinoline C-2), 166.39 (C = O); HRMS (ESI) for C_18_H_16_Cl_2_N_3_O_4_S: calcd 440.0239, found: 440.0237 [M + H]^+^.

### *Ethyl 7-chloro-6-fluoro-4-((3-(N-methylsulphamoyl)phenyl)amino)quinoline-3-carboxylate* (11)

Yellow solid; yield: 40%, ^1^H NMR (DMSO-*d*_6_, 400 MHz) *δ ppm*: 1.07 (*t*, *J* = 6.8 Hz, 3H, CH_2_CH_3_), 2.40 (*s*, 3H, NHCH_3_), 3.90 (*q*, *J* = 6.8 Hz, 2H, CH_2_CH_3_), 7.27 (d, *J* = 7.6 Hz, 1H, H-2 of benzenesulfonamide), 7.41 (*s*, 1H, NHCH_3_), 7.49–7.53 (*m*, 3H, H-4,5,6 of benzenesulfonamide), 8.19 (d, *J* = 11.6 Hz, 1H, H-5 quinoline), 8.26 (d, *J* = 7.2 Hz, 1H, H-8 quinoline), 8.91 (*s*, 1H, H-2 quinoline), 9.79 (*s*, 1H, NH); ^13 ^C NMR (DMSO-*d*_6_, 101 MHz) *δ ppm*: 14.19 (CH_2_CH_3_), 29 (NHCH_3_), 61.52 (CH_2_), 110.18 (quinoline C-5), 110.42 (quinoline C-5), 111.56 (quinoline C-3), 117.44 (benzenesulfonamide C-2), 121.17 (benzenesulfonamide C-4), 121.25 (quinoline C-10), 121.33 (quinoline C-10), 122.63 (benzenesulfonamide C-6), 125.22 (quinoline C-7), 125.43 (quinoline C-7), 130.61 (benzenesulfonamide C-5), 131.58 (quinoline C-8), 140.89 (quinoline C-8), 144.09 (benzenesulfonamide C-3), 146.97 (benzenesulfonamide C-1), 147.02 (quinoline C-9), 147.54 (quinoline C-2), 152.26 (quinoline C-4), 153.76 (quinoline C6), 156.21 (quinoline C-6), 166.24 (C = O); HRMS (ESI) for C_19_H_18_ClFN_3_O_4_S: calcd 438.0691, found: 438.0693 [M + H]^+^.

### CA inhibitory assay

An Applied Photophysics stopped-flow instrument has been used for assaying the CA catalyzed CO_2_ hydration activity^24^. Phenol red (at a concentration of 0.2 mM) has been used as indicator, working at the absorbance maximum of 557 nm, with 20 mM Hepes (pH 7.5) as buffer, and 20 mM Na_2_SO_4_ (for maintaining constant the ionic strength), following the initial rates of the CA-catalyzed CO_2_ hydration reaction for a period of 10–100 s. The CO_2_ concentrations ranged from 1.7 to 17 mM for the determination of the kinetic parameters and inhibition constants. For each inhibitor at least six traces of the initial 5–10% of the reaction have been used for determining the initial velocity. The uncatalysed rates were determined in the same manner and subtracted from the total observed rates. Stock solutions of inhibitor (0.1 mM) were prepared in distilled-deionised water and dilutions up to 0.01 nM were done thereafter with the assay buffer. Inhibitor and enzyme solutions were pre-incubated together for 15 min at room temperature prior to assay, in order to allow for the formation of the E-I complex. The inhibition constants were obtained by non-linear least-squares methods using PRISM 3 and the Cheng-Prusoff equation, as reported earlier^25–29^ and represent the mean from at least three different determinations.

## Results and discussion

### Chemistry

**Scheme 1.**
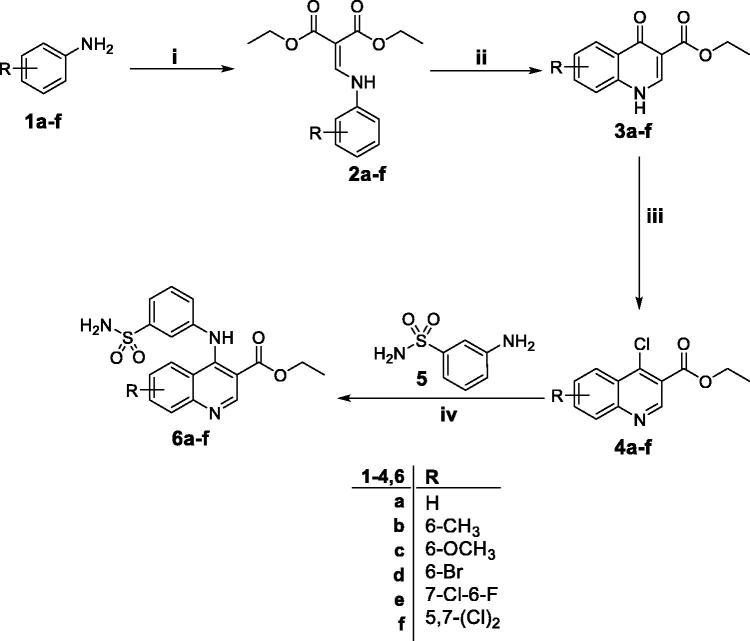
Synthesis of target quinolines **6a–f**; *Reagents and conditions*: (**i**) DEEMM/Ethanol/reflux 1 h; (**ii**) Diphenyl ether/250 °C/45 min; (**iii**) POCl_3_/reflux 1 h; (**iv**) Absolute ethyl alcohol/reflux 4 h.

**Scheme 2.**
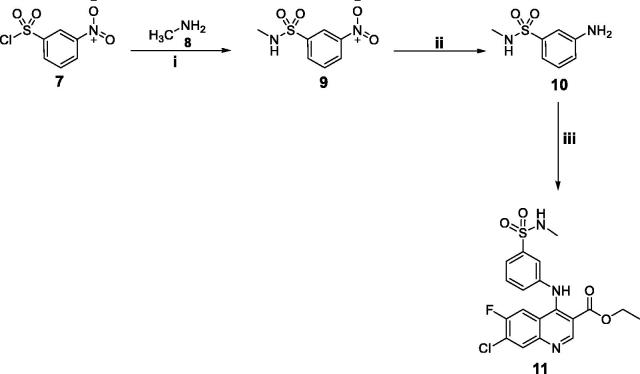
Synthesis of target quinoline **11**; *Reagents and conditions*: (**i**) Hunig's Base/THF/stirring at r.t./1 h; (**ii**) H_2_/10% Pd/C/MeOH/r.t.; (**iii**) Compound **4e**/Absolute ethyl alcohol/reflux 4 h.

The methods adopted for synthesis of the target quinolines **6a**–**f** and **11** are depicted in Schemes 1 and 2. Firstly, anilines **1a**–**f** were heated with diethyl ethoxymethylenemalonate in refluxing ethanol to furnish diethyl 2-((phenylamino)methylene)malonate derivatives **2a**–**f** which thermally cyclised to the corresponding ethyl 4-oxo-1,4-dihydroquinoline-3-carboxylates **3a**–**f** *via* heating in diphenyl ether. Next, chlorination of quinolinones **3a**–**f** was carried out under anhydrous condition through heating with excess of phosphorus oxychloride to afford the key intermediates ethyl 4-chloroquinoline-3-carboxylates **4a**–**f**. The target primary 3-(quinolin-4-ylamino)benzenesulfonamides **6a**–**f** were obtained through a MW assisted nucleophilic substitution reaction of 3-aminobenzenesulfonamide **5** with the appropriate key intermediate **4a**–**f** in ethyl alcohol (Scheme 1).

In Scheme 2, 3-amino-*N*-methylbenzenesulfonamide **10** was prepared as reported earlier^13^ through a nucleophilic substitution for 3-nitrobenzenesulphonyl chloride **7** with methylamine, followed by a catalytic hydrogenation to the nitro function. The later reacted with the key intermediate **4e** in refluxing ethanol to afford the target secondary benzenesulfonamide **11** (Scheme 2).

The structures of the newly prepared quinolines **6a–f** and **11** were confirmed and elucidated by NMR spectroscopy and high resolution mass spectroscopy, which were in full agreement with the postulated structures (Supplementary material).

^1^H NMR spectra of quinolines **6a-f** showed new characteristic signals at *δ* 7.33– 7.37 *ppm*, and 9.68–10.25 *ppm* corresponding to NH_2_ and NH groups, respectively, that distinguished the target quinolines **6a-f** from the key intermediates chloroquinolines **4a-f**. Also, the ^1^H NMR of 7-chloro-6-fluoro-4–(3-methanesulphonylaminophenyamino)-quinoline-3-carboxylic acid ethyl ester (**11**) displayed three significant signals at *δ* 2.99, 9.63 and 9.79 *ppm* assigned to -NHCH_3_, -SO_2_NH- and -NH- protons, respectively.

### Biological evaluation

#### Carbonic anhydrase inhibition

The newly prepared 3-(quinolin-4-ylamino)benzenesulfonamides **6a–f** and **11** were evaluated for their ability to inhibit the physiologically relevant hCA cytosolic isoforms, hCA I and II, by a stopped-flow CO_2_ hydrase assay^24^. The inhibition data of the prepared quinolines and the sulfonamide acetazolamide **AAZ** (as a standard inhibitor) against the two examined isoforms are summarised in Table 1. The following structure-activity relationship (SAR) could be noted regarding the inhibition data reported in Table 1:

**Table 1.** Inhibition data of human CA isoforms hCA I and II for quinolines **6a–f** and **11**, determined by stopped-flow CO_2_ hydrase assay, using acetazolamide (AAZ) as a standard drug.
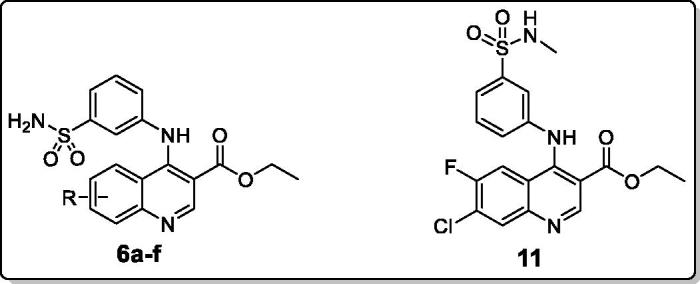

Comp.	R	*K*_I_ (nM)*	
		hCA I	hCA II
**6a**	H	4233.2	223.4
**6b**	6-CH_3_	6644.4	782.3
**6c**	6-OCH_3_	966.0	175.4
**6d**	6-Br	9091.7	3594.8
**6e**	7-Cl-6-F	7604.6	83.3
**6f**	5,7-(Cl)_2_	>10000	>10000
**11**	–	>10000	>10000
**AAZ**	–	250.0	12.0

* Mean from three different assays, by a stopped flow technique (errors were in the range of ± 5–10% of the reported values).

- The secondary sulfonamide reported here (**11**) failed to inhibit the tested hCA isoforms (hCA I and hCA II) up to 10 μM, which confirmed the crucial role of the primary sulfonamide as a zinc-anchoring group, with the additional two hydrogen bonds with Thr199 and Thr200 residues within the enzyme active site.
- The data presented in Table 1 ascribed to the prepared primary sulfonamides (**6a-6e**) weak potency in inhibiting the ubiquitous cytosolic isoform hCA I with inhibition constants (*K*_I_s) in the micromolar range, in detail, between 4.233 and 9.091 μM, except for the 6-methoxy substituted analog **6c** which arose as a submicromolar hCA I inhibitor with a *K*_I_ equals 0.966 μM, which represents 3.8-fold decreased efficacy to the reference drug AAZ (*K*_I_ equals 0.250 μM towards hCA I). On contrary, the 5,7-dichloro substituted primary sulfonamide **6f** failed to inhibit the hCA I up to 10 μM.
  Noteworthy, the SAR outcomes highlighted that grafting the strong electron-donating 6-metoxy group (compound **6c**; *K*_I_ = 0.966 μM) resulted in 4.4-fold efficacy enhancement in comparison to the unsubstituted analogue **6a** (*K*_I_ = 4.233 μM). Regarding the impact of substitution of the quinoline moiety within the primary sulfonamides series **6a-6f**; the inhibitory activities were decreased in the order of 6-OCH_3_ >6-CH_3_ >7-Cl-6-F > 6-Br >5,7-(Cl)_2_.
- The second ubiquitous cytosolic isoform examined here was hCA II. It was apparent from the displayed results (Table 1) that the tested primary sulfonamides (**6a-6e**) effectively interfere with hCA II catalytic activities in submicromolar/micromolar concentration range (*K*_I_ values of 0.083 – 3.594 μM), whereas, no significant inhibition towards hCA II was revealed for quinoline **6f** (*K*_I_ >10 μM). Nevertheless, among the tested quinolines, 7-chloro-6-flouro substituted compound **6e** (*K*_I_ = 0.083 μM) proved to be the most active quinoline in inhibiting hCA II. Moreover, grafting a 6-methoxy group within the quinoline scaffold (compound **6c**; *K*_I_ = 0.083 μM) was advantageous for the inhibitory activity toward hCA II, similarly to the SAR for hCA I inhibition. Regarding the substitution effect for the quinoline moiety; the inhibitory activities towards hCA II were decreased in the order of 7-Cl-6-F > 6-OCH_3_ > 6-CH_3_ > 6-Br > 5,7-(Cl)_2_.

## Conclusion

In summary, we successfully synthesised new benzenesulfonamides, bearing un/substituted ethyl quinoline-3-carboxylate scaffold (**6a-f** and **11**), which were evaluated for their inhibition of hCA I and hCA II. Both the examined isoforms were inhibited by the quinolines reported here in variable degrees; hCA I was inhibited with *K*_I_s in the range of 0.966–9.091 μM, whereas hCA II in the range of 0.083–3.594 μM. Among the tested compounds, the primary 7-chloro-6-flouro substituted sulfonamide derivative **6e** (*K*_I_ = 0.083 μM) proved to be the most active quinoline in inhibiting hCA II, whereas, its secondary sulfonamide analogue **11** failed to inhibit the hCA II up to 10 μM, confirming the crucial role of the primary sulphonamido group, as a ZBG, for CA inhibitory activity.
